# Organizational Principles of the Primate Cerebral Cortex at the Single‐Cell Level

**DOI:** 10.1002/advs.202411041

**Published:** 2025-01-23

**Authors:** Renrui Chen, Pengxing Nie, Liangxiao Ma, Guang‐Zhong Wang

**Affiliations:** ^1^ CAS Key Laboratory of Computational Biology Shanghai Institute of Nutrition and Health University of Chinese Academy of Sciences Chinese Academy of Sciences Shanghai 200031 China

**Keywords:** cell type, cerebral cortex, excitation–inhibition balance, neuron clustering, single‐cell spatial transcriptomics

## Abstract

The primate cerebral cortex, the major organ for cognition, consists of an immense number of neurons. However, the organizational principles governing these neurons remain unclear. By accessing the single‐cell spatial transcriptome of over 25 million neuron cells across the entire macaque cortex, it is discovered that the distribution of neurons within cortical layers is highly non‐random. Strikingly, over three‐quarters of these neurons are located in distinct neuronal clusters. Within these clusters, different cell types tend to collaborate rather than function independently. Typically, excitatory neuron clusters mainly consist of excitatory‐excitatory combinations, while inhibitory clusters primarily contain excitatory‐inhibitory combinations. Both cluster types have roughly equal numbers of neurons in each layer. Importantly, most excitatory and inhibitory neuron clusters form spatial partnerships, indicating a balanced local neuronal network and correlating with specific functional regions. These organizational principles are conserved across mouse cortical regions. These findings suggest that different brain regions of the cortex may exhibit similar mechanisms at the neuronal population level.

## Graphical abstract

This study reveals that neurons in the primate cerebral cortex organize into distinct, non‐random clusters within each cortical layer. Excitatory and inhibitory neurons collaborate in balanced networks, a pattern conserved in mice. These clustered arrangements are linked to specific brain functions, uncovering fundamental principles of brain organization at the single‐cell level.

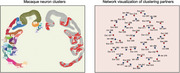

## 1 Introduction

Single‐cell sequencing provides a profound opportunity to explore the state and gene expression profiles of individual cells in the brain, thereby facilitating the inference of their classification, properties, and potential physiological functions.^[^ ^1^, ^2^, ^3^, ^4^, ^5^, ^6^ ^]^ By targeting specific brain regions for sequencing, we can uncover the diversity of cell types within those areas^[^ ^7^, ^8^ ^]^ Moreover, applying single‐cell sequencing across different brain regions and species helps us understand variations in cell type composition among various brain areas.^[^ ^4^, ^5^, ^9^ ^]^ Current research has identified thousands of distinct cell types in the brain.^[^ ^4^, ^5^, ^10^, ^11^, ^12^, ^13^ ^]^ Notably, spatial transcriptome analysis at the single‐cell level further reveals the varied distribution of neuronal cells across different brain regions, significantly enhancing our understanding of the brain's fine‐scale spatial organization.^[^ ^11^, ^14^, ^15^, ^16^, ^17^, ^18^ ^]^ As techniques improve and data accumulate, we anticipate discovering more underlying design principles at the single‐cell level across diverse brain regions.

Large‐scale omics studies of the cerebral cortex have been conducted for years, evolving from microarray techniques to single‐cell sequencing.^[^ ^12^, ^19^, ^20^, ^21^, ^22^ ^]^ Encompassing various developmental stages, distinct cortical locations, and multiple species, these studies have yielded numerous significant discoveries.^[^ ^8^, ^23^, ^24^, ^25^, ^26^ ^]^ Recent studies into the spatial transcriptome of the mouse cortical area have been particularly intriguing, uncovering a complex, spatially distributed cellular architecture.^[^ ^11^, ^14^, ^15^, ^27^, ^28^ ^]^ Within the cortex, neuronal cell types demonstrate spatial gradient effects and engage in complex interactions.^[^ ^11^, ^15^, ^28^ ^]^ Additionally, notable differences in neuronal cell composition across different cortical layers have been documented.^[^ ^16^, ^29^ ^]^ However, there has been limited research on the spatial transcriptome across the entire cerebral cortex of primates, particularly concerning the principles of spatial single‐cell organization of neurons.

In this study, we investigated the spatial distribution of 26 771 303 neuronal cells across the entire cerebral cortex of macaques, examining their fine‐scale spatial organization within different cortical layers. We uncovered a high degree of non‐randomness in the distribution of neurons, resulting in the formation of numerous neuronal clusters. Strikingly, more than three‐quarters of all neurons were located within these spatial clusters. We then analyzed the properties of various neuronal clusters, including their cell type compositions and the interactions between inhibitory and excitatory neuronal clusters. Additionally, we examined the association between the distribution of neuronal clusters and specific functional regions of the brain. Our findings suggest a common mechanism of cortical cell type utilization at the neuronal population level in primates, offering new insights into the micro‐organizational principles of the primate cerebral cortex.

## 2 Results

### 2.1 Neurons in the Primate Cerebral Cortex Cluster Together Within Each Layer

**Figure 1:**
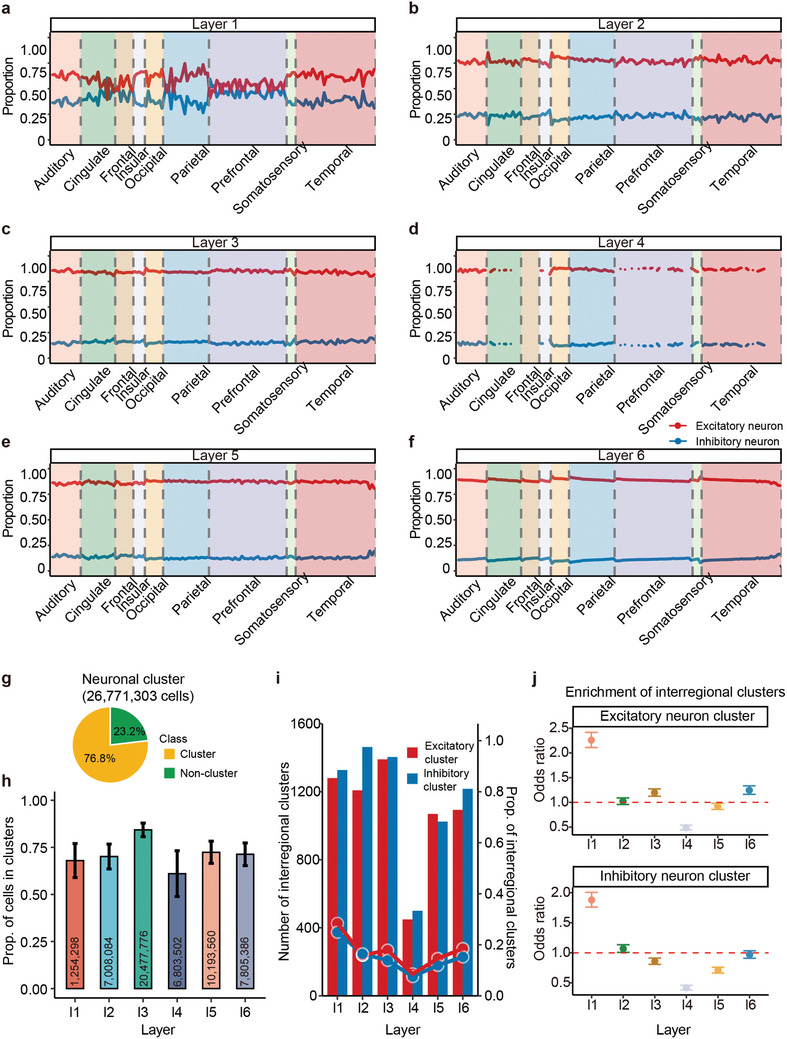
Non‐random distribution of neuron cells within cortical layers of macaques. a–f) Proportions of excitatory neurons (red) and inhibitory neurons (blue) in each layer (Layer 1–6) of 9 major cortical lobes (auditory, cingulate, frontal, occipital, parietal, prefrontal, somatosensory, and temporal). The y‐axis quantifies their proportions, and the x‐axis categorizes the cortical lobes, with dashed lines indicating regional boundaries. Agranular areas, which lack a well‐defined granular layer (Layer IV), are represented as blank. g) The classification of all neurons shows 76.8% in neuronal clusters and 23.2% not in clusters, with a total of 26711303 neuron cells analyzed. h) Proportion of cells in neuronal clusters for layers 1 to 6, showing that a significant number of cells in each layer are part of clusters. The error bars represent the standard error of the mean across 119 Stereo‐seq chips, and the numbers indicate the total neurons analyzed in each layer. i) Interregional clusters exist widely. Bar plot showing the number of interregional clusters detected in each layer, and a line plot showing the proportion of interregional neuronal clusters relative to total clusters in each layer. j) Enrichment of interregional clusters in different layers. The y‐axis indicates the odds ratio, with error bars representing the 95% confidence interval.

Previous research has demonstrated that the ratio of excitatory (E) to inhibitory (I) neurons, known as the E–I ratio, is balanced in layer 2/3 of the mouse cortex.^[^ ^30^, ^31^, ^32^, ^33^ ^]^ However, the status of E–I balance in the cortex of primate brains remains elusive. In this study, we analyzed the distribution of a total of 26771303 neurons across six layers (layers 1–6) in the macaque brain cortex, encompassing 143 brain areas. We calculated the proportion of excitatory neurons to inhibitory neurons in each layer of every brain region. The results, as illustrated in **Figure** **1**a–f, indicate that, except for layer 1, the E–I ratio in layers 2 through 6 generally remains balanced across different brain areas. With an increase in the number of excitatory neurons, there is a gradual increase in the number of inhibitory neurons, demonstrating a highly significant correlation between the two (R > 0.98, P < 1 × 10^−100^, Figure S1a, Supporting Information). Moreover, we observed a significant increase in the E–I ratio from layer 1 to layer 6 (Figure S1b, P < 0.05, Supporting Information), suggesting that in the deeper cortical layers of the primate brain, the proportion of excitatory neurons gradually increases.^[^ ^34^, ^35^ ^]^ The distribution of excitation–inhibition (E–I) ratios in cortical layer 1 is consistent with recent studies demonstrating that the proportion of inhibitory neurons in this layer varies significantly across different species and cortical regions.^[^ ^36^ ^]^

Although the ratio of excitatory to inhibitory neurons is generally balanced across different brain regions, the possibility of small‐scale spatial clustering of excitatory or inhibitory neurons within layers still exists. Such localized clustering could suggest that neuronal populations in these areas perform specialized functions.^[^ ^37^, ^38^, ^39^, ^40^, ^41^, ^42^, ^43^ ^]^ To investigate the non‐randomness in the spatial distribution of neuronal cells, we employed a scanning methodology across each layer using a 1000 µm × 1000 µm window with a step size of 100 µm, documenting the distribution of cell types within each window. We subsequently conducted 10 000 permutation experiments to evaluate the significance of the excitatory and inhibitory neuron distributions within each window. Overlapping windows that significantly enriched the same cell type (either excitatory or inhibitory) were merged into larger areas. This strategy enabled us to determine the presence of regions with significant large‐scale enrichment of either excitatory or inhibitory cells and to further analyze the characteristics of these neuron clusters. Notably, even when employing a finer resolution analysis with a sliding window of 400 µm × 400 µm and a 100 µm step size, the primary conclusions of our study remained consistent.

We observed that the size of spatial neuron clusters varied from a few cells to several thousand, with a median of 96 cells (Table S1, Supporting Information). Notably, out of the 26 771 303 neurons we examined, 20 574 158 were found within these clusters. This indicates that, overall, more than three‐quarters (76.8%) of the neurons were clustered together (Figure  1g). In each layer, the majority of cells were also located within clusters (Figure  1h). The highest proportion of cells within clusters was observed in layer 3, at 84.25% (Figure  1h), suggesting that neurons in layer 3 are more prone to clustering, which indicates a denser and more interconnected neuronal architecture in this layer.^[^ ^44^, ^45^ ^]^ Conversely, the lowest proportion of cells within clusters was found in layer 4, at 61.03% (Figure  1h). Regionally, the occipital and frontal areas exhibited a higher tendency for clustering, particularly enriched in excitatory neurons (P = 0.012 and 0.0058, Wilcoxon rank‐sum test), with rates of 78.16% and 77.37%, respectively. In clusters predominantly composed of inhibitory neurons, the cingulate area showed a higher degree of clustering, at 77.69% (P = 0.004, Wilcoxon rank‐sum test).

Each of the 143 subcortical regions is spanned by at least one neuronal cluster, with 15.63% of these clusters being interregional (Figure  1i). Our analysis revealed that interregional clusters typically contain more cells compared to intraregional clusters (P < 1 × 10^−100^), a trend consistent across all six layers. In clusters of excitatory neurons, inter‐regional clusters are more prevalent in layer 1 (p = 1.22 × 10^−100^), layer 3 (p = 1.92 × 10^−8^), and layer 6 (p = 7.8 × 10^−10^, Figure  1j). Conversely, in clusters of inhibitory neurons, inter‐regional clusters are more likely to be found in layer 1 (p = 1.59 × 10^−73^) and layer 2 (p = 0.028, Figure  1j). Additionally, at the lobe level, numerous clusters were found spanning two cerebral lobe areas. These findings suggest that traditional divisions of brain subregions may be reconsidered in light of fine‐scale structural analyses, revealing a more complex micro‐organization within the brain.

### 2.2 Excitatory and Inhibitory Neuron Clusters Exhibit Significant Differences in Properties and Cell Type Usage

**Figure 2:**
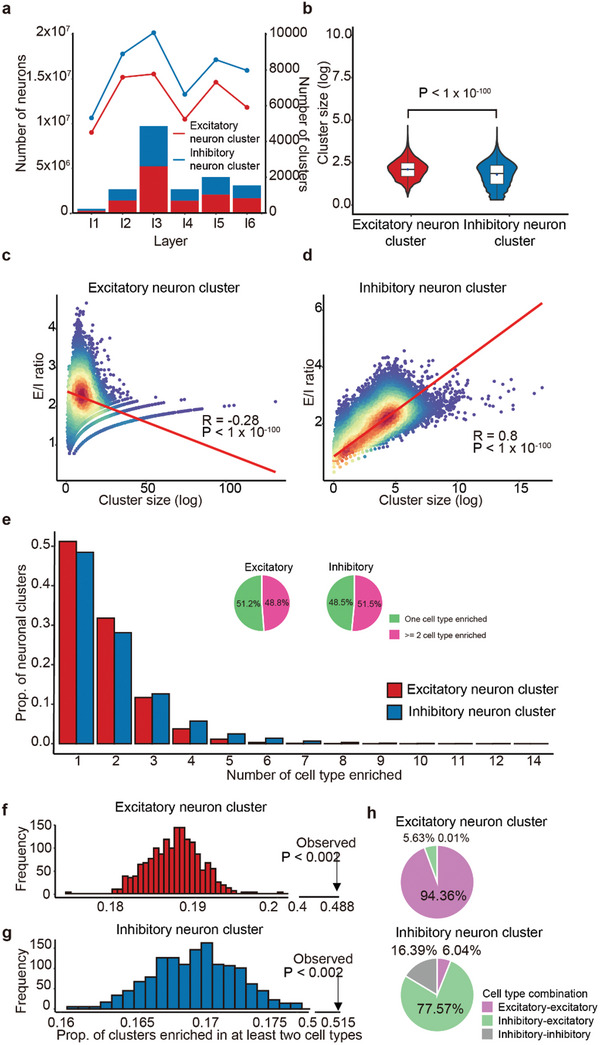
Properties and cell type usage of excitatory and inhibitory neuron clusters. a) Neuron cell number and cluster distribution by cortical Layer. The bar plot illustrates that the neuron numbers in excitatory neuron clusters (red) and inhibitory neuron clusters (blue) across cortical layers 1 to 6 are similar. The line plot shows that the number of inhibitory neuron clusters (blue) is higher than that of excitatory neuron clusters (red) in each layer. b) Comparison of cluster sizes of excitatory neuron clusters (red) and inhibitory neuron clusters (blue), using the Wilcoxon rank‐sum test. c,d) Correlation analysis of the sizes of excitatory and inhibitory neuron clusters with their E/I ratios. A scatter plot of the excitatory neuron cluster shows a negative correlation between cluster size (log scale) and E/I ratio, while the inhibitory neuron cluster exhibits a strong positive correlation between cluster size (log scale) and E/I ratio. Significance was tested using the Pearson correlation method. e) The enrichment of cell types in neuronal clusters. Bar plots show the proportion of cell types enriched in excitatory and inhibitory neuron clusters. Pie charts represent the proportions of neuron clusters enriched with single or multiple cell types, indicating that nearly half of the clusters are enriched with multiple cell types. f,g) Permutation test to determine the significant level of neuron clusters enriched in multiple cell types. Histograms illustrate the proportion of at least two cell types enriched in excitatory (f) or inhibitory (g) neuron clusters in the permutation experiment. These figures show that the proportion of multiple cell types enriched in a neuronal cluster is significantly higher than expected (P < 0.002). h) Cell type usage in excitatory and inhibitory neuron clusters. Pie charts display the percentages of different cell type combinations within excitatory and inhibitory neuron clusters, highlighting the distinct patterns of cell‐type co‐occurrence.

We observed significant differences between excitatory and inhibitory neuron clusters. Firstly, although inhibitory neurons constitute approximately 15% of all neuronal cells, the number of neuronal cells within inhibitory neuron clusters is similar to the number of neuronal cells within excitatory neuron clusters (10 703 572 vs 11 964 674 cells), and this holds true for each layer **Figure**  **2**a, Table S1, Supporting Information). Thus, the presence of neuronal cells in inhibitory neuron clusters is significantly higher than expected (P < 1 × 10^−100^, Chi‐square test). Additionally, the number of inhibitory neuron clusters significantly exceeds that of excitatory neuron clusters in each layer (Figure  2a). The size of inhibitory neuron clusters is also significantly smaller than that of excitatory neuron clusters (Figure  2b). A significant negative correlation was found between the size of excitatory neuron clusters and their E–I ratios (r = −0.28, P < 1 × 10^−100^, Figure  2c), whereas a significant positive correlation was observed between the size of inhibitory neuron clusters and their E–I ratios (r = 0.80, P < 1 × 10^−100^, Figure  2d). These findings suggest distinct electrophysiological properties for these two types of clusters. Moreover, the average distance between cells within excitatory neuron clusters (44.88 µm ± 22.36 µm) is significantly shorter than that within inhibitory neuron clusters (50.38 µm ± 34.50 µm, P = 1.1 × 10^−123^), indicating a higher neuronal density within excitatory neuron clusters. Intriguingly, we also identified 3893 excitatory neuron clusters that did not contain any inhibitory neurons, with an average of 22 cells per cluster; and 179 inhibitory neuron clusters without any excitatory neurons (≥ 5 cells on average). These clusters may play specialized functions in the cortex.

The composition of different cell types within neuron clusters may reflect their distinct electrophysiological properties. To explore whether excitatory and inhibitory neuron clusters exhibit unique patterns of cell type usage, we first randomized the coordinates of each cell type among excitatory and inhibitory neurons and counted the number of each cell type in each cluster. This experiment was repeated 10 000 times to calculate the statistical significance of each cell type's enrichment. Our results indicated that cell types generally tend to cluster together rather than randomly. In nearly half of the neuronal clusters (48.8% for excitatory neuron clusters and 51.5% for inhibitory neuron clusters), two or more cell subtypes significantly coexisted (Figure  2e, Table S2, Supporting Information). These ratios are significantly higher than expected by chance, suggesting that different neuronal cell types are more likely to collaborate rather than function independently (Figure  2f,g and P < 0.002). To further understand the co‐occurrence among cell types, we analyzed all clusters enriched with multiple cell types. We found that more than 90% of excitatory neuron clusters consisted of combinations of excitatory‐excitatory cells (Figure  2h), while over 75% of inhibitory neuron clusters were composed of inhibitory‐excitatory cell types (Figure  2h). Similar trends were observed for clusters enriched with only two cell types, with excitatory‐excitatory pairs accounting for over 95% in excitatory neuron clusters, and excitatory‐inhibitory combinations comprising over 60% in inhibitory neuron clusters. These findings demonstrate that excitatory and inhibitory neuron clusters employ different strategies for cell type usage.

We also discovered that adjacent clusters tend to utilize similar cell types. By analyzing the similarity in cell type compositions between adjacent neuron clusters of the same type and comparing these with non‐adjacent neuron clusters within the same layer, we observed that both excitatory and inhibitory neuron clusters exhibited significantly higher similarity among adjacent clusters than among non‐adjacent ones (Figure S2a, P = 2.02 × 10^−198^ and 4.4 × 10^−143^, respectively, Supporting Information). This observation was also corroborated by analyses using both the Pearson and Spearman correlation methods (Figure S2b,c, P < 1 × 10^−40^ in all the analyses, Supporting Information). The pronounced similarity in cell type usage between neighboring clusters may facilitate enhanced communication among them, potentially influencing overall neural network dynamics.

### 2.3 The Majority of Excitatory and Inhibitory Neuronal Clusters Form Partnerships

**Figure 3:**
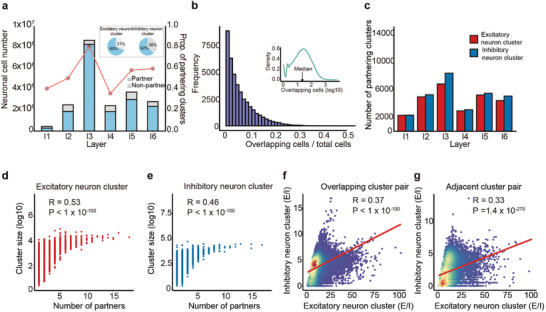
Partnership between excitatory and inhibitory neuron clusters. a) The majority of excitatory and inhibitory neuron clusters have partners in each layer. A bar plot shows the number of neuronal cells in a partnership and the number of neuronal cells without a partnership. Additionally, a line plot displays the proportion of partnering clusters in each layer, and a pie chart represents the proportion of partnering clusters among all clusters for both excitatory and inhibitory neuron clusters, respectively. b) The overlap between partnering clusters is relatively low. A histogram depicts the ratio of overlapping cells to total cells per partnered cluster, with a density plot showing the distribution of shared neurons. c) Inhibitory neuron clusters tend to have more partners than excitatory neuron clusters. A bar plot shows the number of partner clusters for excitatory (red) and inhibitory (blue) neurons. d,e) Correlation of the cluster size with the number of partners. Scatter plots for excitatory (d) and inhibitory (e) neuron clusters exhibit a strong positive correlation between cluster size (log scale) and the number of partners. Significance was tested using the Pearson correlation method. f,g) The scatter plots for overlapping (f) and adjacent (g) cluster pairs reveal a similar positive correlation between the E/I ratio of the partners. Significance was tested using the Pearson correlation method.

Next, we explored the spatial relationship between adjacent excitatory and inhibitory neuron clusters by analyzing the overlap of cells between them, which may indicate a unique partnership. Specifically, we found that approximately 70% of excitatory neuron clusters and 62% of inhibitory neuron clusters are partnered (**Figure**  **3**a). This ratio remains high across all layers (Figure  3a, Figure S3, Supporting Information). Between these partners, there's a median of 21 shared neurons. However, the overlap of cells between them is generally low, typically encompassing less than 50% of the total cells within any specific cluster (Figure  3b and Table S3, Supporting Information). Inhibitory neuron clusters tend to have more partners than excitatory neuron clusters (P = 0.018, Figure  3c). Both types of clusters exhibit a significant positive correlation between cluster size and the number of partners (R > 0.5, p < 1 × 10^−100^, Figure  3d,e), but there is no significant relationship between the sizes of the partnering clusters. These results suggest that larger neuronal populations tend to maintain balanced relationships through more partners, facilitating diverse interactions within the neural network.

Interestingly, we discovered a significant positive correlation between the E–I ratios of coupled excitatory and inhibitory neuron clusters (r = 0.37, P < 1 × 10^−100^, Figure  3f). Notably, excitatory neuron clusters with low E–I ratios were more likely to form partnerships (r = −0.16, P = 1.4 × 10^−143^), whereas inhibitory neuron clusters with high E–I ratios tended to have partners (r = 0.22, P < 1 × 10^−100^). Furthermore, among the 3893 clusters containing only excitatory neurons, significantly fewer partners were detected (p = 3.3 × 10^−156^), suggesting that these clusters tend to work independently. Even in the absence of direct overlap, the E–I ratios between adjacent excitatory‐inhibitory cluster pairs showed a significant positive correlation (r = 0.33, P< 1 × 10^−100^, Figure  3g). This suggests that the physical proximity between clusters has physiological significance. Collectively, these findings provide crucial insights into the functional organization of the neocortex at the neuronal population level.

### 2.4 Conserved Pattern of Neuronal Clusters in Mouse Cortical Layers Were Observed

**Figure 4:**
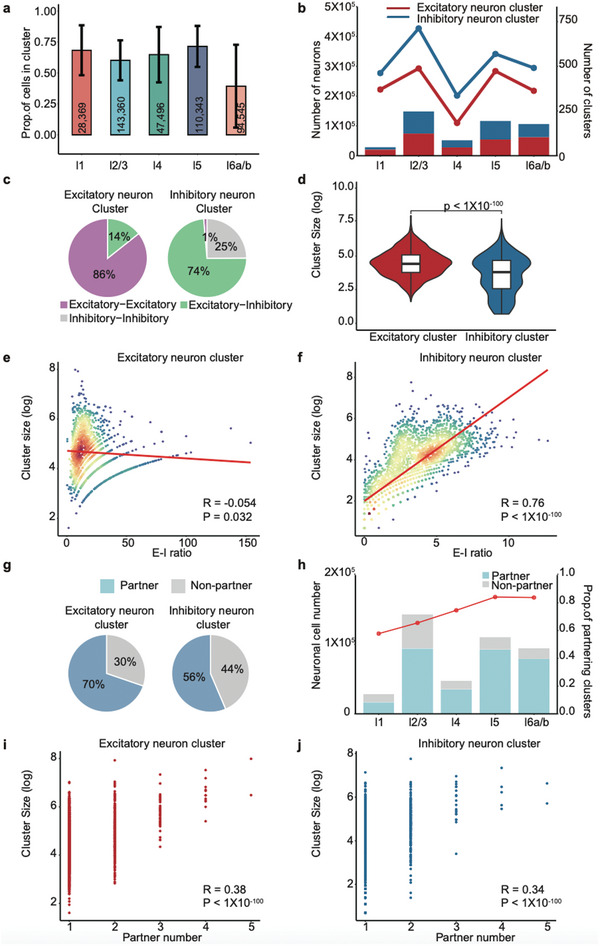
Non‐random distribution of neuronal cells across cortical layers in the mouse. a) Proportion of cells within neuronal clusters from layer 1 to layer 6. Error bars represent the standard error of the mean, and numbers above each bar indicate the total number of neurons analyzed per layer. b) Number of neuronal clusters in each layer, comparing inhibitory neuron clusters (blue) and excitatory neuron clusters (red); inhibitory clusters are more numerous overall. c) Comparison of cluster sizes for excitatory (red) and inhibitory (blue) neuron clusters, with statistical significance determined via the Wilcoxon rank‐sum test. d) Pie charts showing the relative frequencies of distinct cell‐type combinations within excitatory and inhibitory neuron clusters, highlighting their unique co‐occurrence patterns. e,f) Scatter plots of cluster size (log‐scale) versus E–I ratio. Excitatory neuron clusters (e) display a negative correlation, whereas inhibitory neuron clusters (f) show a strong positive correlation. Significance was assessed using Pearson correlation. g) Most excitatory and inhibitory neuron clusters in each layer form partnerships. Pie charts illustrate the proportion of partnering clusters among all excitatory or inhibitory neuron clusters. h) Bar plot showing the number of neuronal cells involved in partnerships between excitatory and inhibitory neuron clusters compared to those without partnerships. i,j) Scatter plots illustrating the relationship between cluster size (log‐scale) and the number of partners in excitatory (i) and inhibitory (j) neuron clusters. Significance was assessed using Pearson correlation.

Utilizing spatial transcriptomic data from the mouse,^[^ ^15^ ^]^ we analyzed the distribution of 632 144 neurons across the six layers of the mouse cortex. Consistent with observations in the monkey cortex, the majority of neurons within each cortical layer are organized into distinct clusters (**Figure**  **4**a). Similarly, each layer contains a significantly higher number of inhibitory neuron clusters compared to excitatory ones (Figure  4b), with inhibitory clusters also being smaller in size (Figure  4c). Examination of cell subclass enrichment revealed that over 86% of excitatory clusters are composed of excitatory–excitatory cell combinations, while more than 74% of inhibitory clusters consist of inhibitory–excitatory cell types (Figure  4d). This indicates that excitatory and inhibitory neuron clusters employ different strategies for cell type composition, aligning with findings in monkey studies. Additionally, the size of excitatory clusters is significantly negatively correlated with their excitation‐inhibition (E–I) ratio (r = −0.054, p = 0.032, Figure  4e), whereas inhibitory cluster size shows a significant positive correlation with E–I ratio (r = 0.76, p < 1 × 10⁻¹⁰⁰, Figure  4f), suggesting distinct electrophysiological properties between the two cluster types. Spatial relationships between adjacent excitatory and inhibitory clusters were further investigated by analyzing cell overlap, revealing that approximately 70% of excitatory and 56% of inhibitory clusters form partnerships (Figure  4g,h). Both excitatory and inhibitory clusters exhibit significant positive correlations between cluster size and the number of partner clusters (excitatory: R = 0.38, p = 8.74 × 10⁻⁴⁶; inhibitory: R = 0.34, p = 2.73 × 10⁻⁴⁰, Figure  4i,j). These results suggest that larger neuronal clusters engage with more partners to maintain balanced interactions within the neural network in the mouse cortex as well.

### 2.5 The Distribution of Neuronal Clusters Correlates with the Physiological Properties of Brain Regions

**Figure 5:**
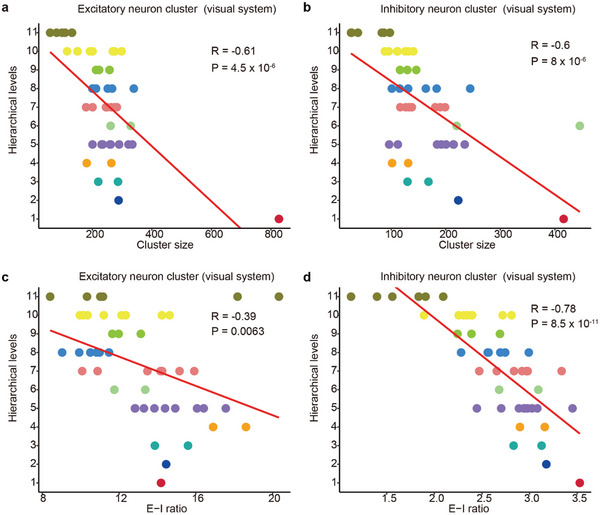
Both cluster size and the E/I ratio are negatively correlated with the cortical hierarchy in the primate visual system. a,b) Association between cluster size distribution and cortical hierarchy of the primate visual system. Scatter plots show the correlation between hierarchical levels and cluster sizes for both excitatory (a) and inhibitory neuron clusters (b), indicating that larger neuron clusters are located in early processing pathways. Significance was tested through Pearson correlation. c,d) Association between E–I ratio distribution and cortical hierarchy of the primate visual system. Scatter plots show the correlation between hierarchical levels and E–I ratio for both excitatory (c) and inhibitory neuron clusters (d), indicating that larger neuron clusters are in early processing pathways. Significance was tested through Pearson correlation.

We explored whether the identified neuronal clusters are associated with specific physiological functions, focusing on brain regions related to the primate visual system,^[^ ^46^ ^]^ somatosensory system,^[^ ^46^ ^]^ and the default mode network (DMN).^[^ ^47^ ^]^ Initially, we observed that within the primate visual system, regions involved in early processing pathways tend to utilize larger neuronal clusters, whereas regions engaged in higher‐level pathways prefer smaller clusters. This negative correlation between the hierarchical level and cluster size was observed in both excitatory and inhibitory neuron clusters (**Figure**  **5**a,b and R = −0.6, P < 1 × 10^−5^ for both datasets). Additionally, we discovered that early visual pathways exhibit a higher E–I ratio (Figure  5c,d). In the somatosensory system, excitatory neuron clusters demonstrated a similar pattern (R = −0.55, p < 0.01), further underscoring the importance of large clusters in the early stages of information processing. These findings reveal a close relationship between the distribution of neuronal clusters and the levels of information processing in which they are involved.

**Figure 6:**
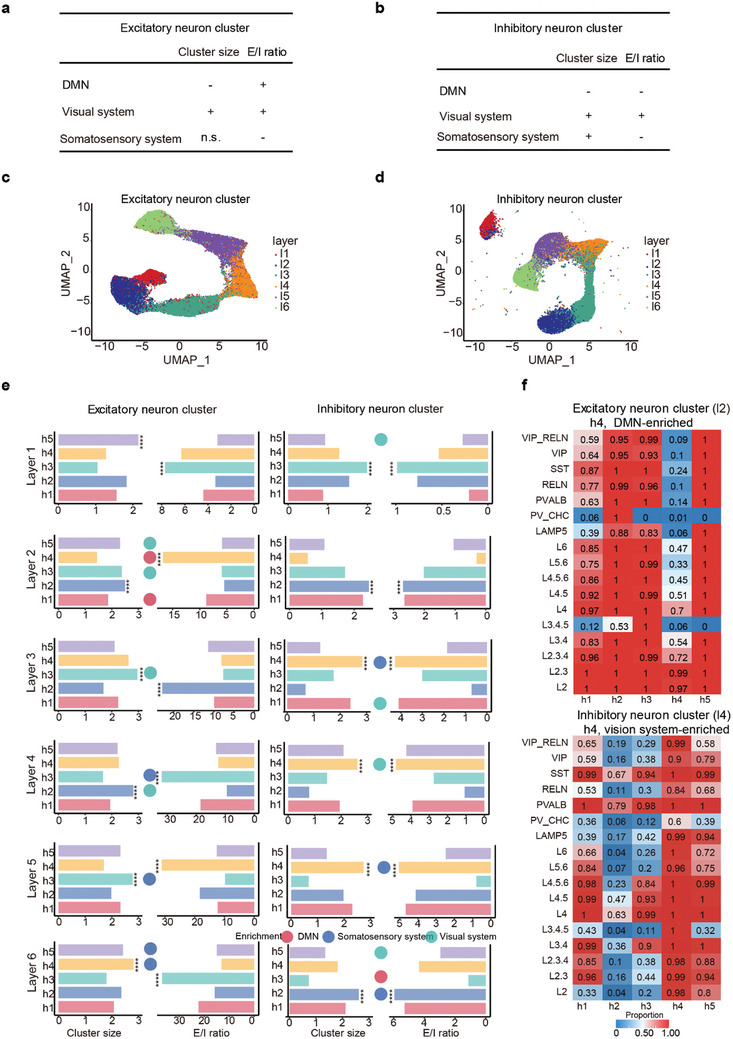
The linkage between neuronal clusters and physiological properties of brain regions. a,b) A table summarizes the relationship between the distribution of excitatory and inhibitory neuron clusters and different functional brain regions, including DMN, visual system, and somatosensory system, highlighting their function‐specific distributions. + positive correlation with p < 0.05, – negative correlation with p < 0.05, n.s., not significant. c,d) UMAP visualizations of excitatory neuron clusters (c) and inhibitory neuron clusters (d) display the diverse spatial distribution of these clusters across different cortical layers. e) Bar plots illustrate the distribution of cluster sizes and E/I ratios across different groups, while bubble plots show their degree of enrichment in the DMN, visual, and somatosensory systems. f) Distinct cell type composition for different groups. The top heatmap shows excitatory neuron clusters for layer 2, with group h4 being DMN‐enriched. The bottom heatmap shows inhibitory neuron clusters for layer 4, with group h4 being visual system‐enriched. In the heatmaps, values closer to 1 indicate the widespread presence of the cell type in this group.

In the brain regions associated with the DMN, both excitatory and inhibitory neuron clusters were found to be significantly smaller (p = 1.54 × 10^−17^ and 1.27 × 10^−9^, respectively, Wilcoxon rank‐sum test, **Figure**  **6**a,b). In contrast, within the primate visual system, the sizes of both excitatory and inhibitory neuron clusters were significantly larger than those in other brain regions (p = 3.69 × 10^−57^ and 0.00047, respectively, Wilcoxon rank‐sum test, Figure  6a,b). Regarding the E–I ratio, this value was generally higher in the primate visual system compared to other brain regions (p = 2.56 × 10^−26^ and 2.82 × 10^−55^, respectively, Wilcoxon rank‐sum test). However, in the somatosensory system, the E–I ratio was lower than in other regions (P < 1 × 10^−5^ in both comparation, Wilcoxon rank‐sum test, Figures  5, 6). For the DMN, the E–I ratio of excitatory neuron clusters was higher than in other brain regions (p = 9.23 × 10^−7^), while the E–I ratio of inhibitory neuron clusters was lower than in other regions (p = 2.82 × 10^−9^, Figure  6a,b). These findings indicate that different functional regions of the brain preferentially utilize neuronal clusters with distinct characteristics, reflecting the brain's specialized strategies for processing various types of information.

Next, we investigated whether neuronal clusters with specific cell type combinations are associated with particular functional regions. Initially, we conducted a clustering analysis on the cellular composition of these neuronal clusters. Our findings revealed that both excitatory and inhibitory neuron clusters could be distinctly classified according to cortical layers 1–6 (Figure  6c,d), demonstrating significant variations in their cell type composition across different layers. To further refine our classification of these neuronal clusters, we employed hierarchical clustering to analyze them within each individual layer.

For excitatory neuron clusters, we identified two groups associated with DMN enrichment, four groups related to the visual system and another four groups linked to the somatosensory system (adjusted P < 0.05, Figure  6e, Figure S4, Supporting Information). Each of these groups exhibited distinct cell type compositions. In the DMN‐enriched groups, within layer 2, we identified a group, DMN‐l2‐Exh4, characterized by a high frequency of L2 and L2.3 cell types and a minimal occurrence of L3.4.5 and inhibitory neuron cell types (Figure  6f). This group also exhibited a relatively high E–I ratio. In layer 6, we identified a cluster, DMN‐l6‐Exh3, characterized by the absence of L3.4.5 cell types among excitatory neurons, with lower frequencies of L2, L2.3, L2.3.4, and L3.4 cell types, and almost no presence of inhibitory cell types. This cluster also displayed a relatively high E–I ratio. In the somatosensory system‐enriched groups, within layer 5, we identified a group, SOMA‐l5‐Exh3, characterized by the presence of all cell types, with the highest frequency of PV_CHC cells (Figure S3a, Supporting Information), and displaying a relatively low E–I ratio. In layer 6, we found two groups, SOMA‐l6‐Exh4 and SOMA‐l6‐Exh5, both exhibiting comparable E–I ratios and containing all types of excitatory neurons. In the vision system‐enriched groups, within layer 2, we identified two clusters, VIS‐l2‐Exh3 and VIS‐l2‐Exh5, both lacking PV_CHC cells. In layer 3, we found a cluster named VIS‐l3‐Exh4, characterized by the absence of PV_CHC cells but the presence of other cell types (Figure S3b and Table S4, Supporting Information).

For inhibitory neuron clusters, we identified one group associated with DMN enrichment and four groups related to the visual system (adjusted P < 0.05, Figure  6e, Table S4, Supporting Information). In the visual system‐enriched groups, in layer 4, we identified a group named VIS‐l4‐Inh4, characterized by the widespread presence of cell types and a relatively high E–I ratio (Figure  6f). In layer 1, we found VIS‐l1‐Inh1, characterized by the universal presence of RELN and relatively low E–I ratios. In layer 3, we identified a group named VIS‐l3‐Inh1, characterized by the presence of all cell types except PV_CHC cells, with a relatively low frequency of L3.4.5 cells (Figure S3c, Supporting Information). Within the DMN‐enriched groups, at layer 6, we identified a group named DMN‐l6‐Inh3, characterized by a generally low frequency of most cell types and a relatively low E–I ratio (Figure S3d, Table S4, Supporting Information). These findings suggest that neuronal clusters with specific combinations of cell types indeed tend to appear in specific functional regions, reflecting how the brain adapts to complex information processing demands through diverse cell type configurations.

## 3 Discussion

By analyzing the single‐cell spatial transcriptome of millions of neurons across layers 1 to 6 of the cerebral cortex in macaque monkeys, we observed a highly non‐random spatial distribution of these neurons within the cortical layers. Over three‐quarters of the neurons were located within these clusters. Notably, different neuron cell types tend to collaborate rather than function independently. Excitatory neuron clusters and inhibitory neuron clusters follow distinct cell type combination rules; excitatory neuron clusters are primarily composed of excitatory cells, while inhibitory neuron clusters predominantly consist of a mix of excitatory and inhibitory cell types. More importantly, the majority of excitatory and inhibitory neuron clusters tend to form partnerships with each other. These patterns are strongly conserved in the mouse cortex. Finally, the spatial clustering of neurons was closely associated with the physiological functions of specific brain regions.

The spatial clustering of neurons in each cortical layer offers intriguing insights. Firstly, the total number of neurons within excitatory and inhibitory neuron clusters is roughly equivalent, indicating a balanced neuron network in this aspect.^[^ ^48^, ^49^, ^50^, ^51^ ^]^ Secondly, there is considerable variability in the cell type composition of both excitatory and inhibitory neuron clusters, with marked differences in cluster size and the E–I ratio observed. This variability suggests that each neuron cluster is context‐dependent, and potentially linked to unique electrophysiological functions.^[^ ^52^, ^53^ ^]^ Typically, excitatory neuron clusters are composed predominantly of excitatory neurons, whereas inhibitory neuron clusters feature a heterogeneous mix of both inhibitory and excitatory cell types. These two cluster types may serve distinct functions, with excitatory neurons, such as pyramidal cells, projecting to other cortical and subcortical areas,^[^ ^34^ ^]^ while inhibitory neurons contribute to the modulation of local circuits.^[^ ^35^, ^54^ ^]^ Much of the existing research exploring the interplay between inhibitory and excitatory neurons has predominantly focused on inhibitory clusters, examining the roles of mixed inhibitory‐excitatory cell types. Further investigation into the physiological functions of purely excitatory cell types in excitatory neuron clusters is warranted. Thirdly, excitatory and inhibitory neuronal clusters may differ in their fundamental electrophysiological properties; excitatory clusters exhibit higher activity in neural signaling and effectively form fine‐scale network communications,^[^ ^55^ ^]^ whereas inhibitory clusters can shape excitatory circuits in a context‐specific manner.^[^ ^56^ ^]^ This spatial clustering may facilitate rapid synchronization across local neural networks.

The observation that the vast majority of excitatory and inhibitory neuron clusters form partnerships underscores the need for balance between excitatory and inhibitory clusters at the neuronal population level,^[^ ^48^, ^50^, ^51^, ^57^ ^]^ as they collaborate to maintain the stability of neural networks.^[^ ^51^ ^]^ This explains why larger clusters require a greater number of partners. These larger clusters may be involved in more complex information processing tasks, necessitating a more intricate balance and robust feedback through specific combinations and interactions of neuronal cells. Furthermore, the coupled excitatory and inhibitory neuron clusters, as well as adjacent clusters, are likely to co‐evolve for information processing during evolution. This fine‐grained structure may be crucial for accurately modeling local neuronal activity at the population level.^[^ ^48^, ^49^, ^58^ ^]^

Neurons demonstrate significant plasticity during cortical development, including experience‐dependent plasticity.^[^ ^59^, ^60^, ^61^ ^]^ We propose that this plasticity extends to spatial neuronal clusters, both developmentally and evolutionarily. Such plasticity encompasses changes in cluster size and cellular composition, which are crucial for optimizing and integrating information. Additionally, the interconnections between different neuronal clusters, including those between coupled excitatory‐inhibitory clusters and adjacent clusters, may also be dynamically regulated. This plasticity illustrates how the brain responds to new information by modifying the architecture of local neural networks,^[^ ^62^, ^63^ ^]^ highlighting the brain's capacity to reorganize itself in response to changing environmental demands.

Each gene possesses a unique function, despite many exhibiting high sequence similarities within the genome. Genes have also displayed signatures of variation due to genetic drift and natural selection.^[^ ^64^, ^65^, ^66^, ^67^ ^]^ Similarly, neuronal clusters may exhibit these properties. In the future, a crucial research direction will involve exploring the electrophysiological properties and functions of various spatial neuronal clusters. This raises several critical questions: Are there neuronal clusters essential for human brain cognition? Have certain neuronal clusters undergone rapid evolutionary changes? Do some neuronal clusters play significant roles in specific brain functions in primates and are responsible for particular behaviors? Ultimately, our goal is to annotate functional terms for each neuronal cluster, akin to gene functional annotations in Gene Ontology (GO) or KEGG analysis.^[^ ^68^, ^69^ ^]^ This is essential for advancing our understanding of the systematic mechanisms governing cell populations in the brain.

In summary, our analysis of extensive single‐cell spatial transcriptome data across the entire cerebral cortex of macaques has revealed a significant non‐random distribution of neural cells within cortical layers. Additionally, we observed an enrichment of diverse cell types within both excitatory and inhibitory neuron clusters, including notable partnerships between these clusters. These results indicate that the neuronal network is balanced both globally and locally. We also note that these organizational principles may have significant implications for understanding the computational architecture of the brain. Due to current technical limitations, we are unable to obtain large‐scale morphological and specific circuit architecture data regarding these clusters. In the future, acquiring such data will enable a better understanding of their functional significance. These discoveries shed light on the spatial organizational principles of neurons and their underlying mechanisms of organization across various layers, thereby providing a crucial foundation for modeling cortical neuron distributions at the single‐cell level.

## 4 Experimental Section

### Stereo‐Seq Data Preprocessing

A total of 119 coronal sections from the same monkey, each at different coronal coordinates, were downloaded,^[^ ^17^ ^]^ which were carefully processed. Briefly, the macaque cortex was first divided into 143 regions, including areas such as prefrontal, frontal, cingulate, somatosensory, and others, using the Saleem and Logothetis (2012) atlas.^[^ ^70^ ^]^ For each coronal section, cortical regions and laminar boundaries were manually traced based on mRNA expression patterns, nuclear staining, and NeuN immunostaining. To align the sections with anatomical coordinates, the D99 macaque atlas (https://afni.nimh.nih.gov/pub/dist/doc/htmldoc/nonhuman/macaque_tempatl/atlas_d99v2.html) was used. The sections were manually rotated until they were aligned, then assembled to create an initial 3D brain reconstruction, which was further aligned to the D99 atlas using affine transformation. Finally, all transformations were applied to the spatial transcriptomics data, mapping individual cell coordinates to the atlas. The coordinates of each cell were extracted to map the spatial distribution within the cortical layers. Only the positional information of neurons, including 22739334 excitatory (glutamatergic) neurons and 4031969 inhibitory (GABAergic) neurons were retained. These neurons are distributed across 143 brain regions, including prefrontal, frontal, cingulate, somatosensory, insular, auditory, temporal, parietal, occipital, and piriform areas. Each brain region contains neuronal cell location data from layer 1 to layer 6.

### Identification of Excitatory and Inhibitory Neuron Clusters

To investigate the non‐random distribution of neurons within cortical layers, a window scanning approach was employed using 1000 µm x 1000 µm windows with a step size of 100 µm, recording the distribution of cell types within each window. Subsequently, the coordinates of each cell type within each layer were randomly shuffled and this randomization experiment was repeated 10 000 times to evaluate the statistical significance of excitatory and inhibitory cell types within each window (p < 0.05). For windows showing significant distributions of the same cell type (excitatory or inhibitory), windows were merged with overlapping cells together. Further the main conclusions of the manuscript were validated using a sliding window of 400 µm x 400 µm with a step size of 100µm. For the mouse dataset, a sliding window analysis was employed using windows sized 400 µm × 400 µm with a step increment of 20 µm. To assess the statistical significance of the observed distributions of excitatory and inhibitory neurons, the spatial coordinates of each cell type were randomly shuffled within their respective cortical layers, and this randomization process was repeated 10 000 times.

### Identification of Cell Type Co‐Occurrence in Neuron Clusters

To investigate whether different cell types tend to co‐occur in a neuronal cluster, the coordinates of each cell subtype within each layer were shuffled and this randomization experiment was repeated 10 000 times. Then the significance of the cell type usage in both excitatory and inhibitory neuron clusters (p < 0.05) was assessed. The two cell types were considered to co‐occur within a given cluster if both show significant enrichment in this cluster. The whole process was repeated 500 times to evaluate whether the number of neuron clusters enriched with multiple cell types was significantly higher than expected.

### Calculating the Average Distance Between Cells Within Neuron Cluster

To calculate the average distance (µm) between cells within clusters, the KD‐tree search algorithm^[^ ^71^ ^]^ was employed to find the nearest neighbor for each cell. This algorithm significantly reduces computational time. The Euclidean distance between cells was calculated, and the average distance within each cluster was determined by averaging the shortest distances between all cell pairs within the cluster.

### Calculation of Distances Between The Neighboring Excitatory and Inhibitory Neuron Clusters

The KD‐tree algorithm was used to search for the nearest pair of cells between excitatory and inhibitory neuron clusters within cortical layers and calculated their Euclidean distance as the shortest distance. For each cluster, its closest neighboring cluster was identified.

### Identification of Partnerships Between Excitatory and Inhibitory Neuron Clusters

If there are shared cells between excitatory and inhibitory neuron clusters, these two clusters were defined as being in a partnership. It is observed that the majority of these cluster partnerships involve an overlap of more than one cell, with a median of 21 overlapping cells.

### Calculation of E–I Ratio for Each Cluster

For each neuronal cluster, the E–I ratio was calculated by determining the ratio of the number of excitatory cells to inhibitory cells within the cluster. Significant variations in this ratio are observed across different clusters.

### Calculating the Similarity of Cell Type Usage in Adjacent Clusters

To determine whether adjacent clusters tend to utilize similar cell types, the proportion of each neuron subclass within each cluster was computed. Then the `cor.test` function in R was employed to assess the similarity in cell type usage patterns between neighboring clusters. For partnering clusters, the Jaccard coefficient was calculated by dividing the number of overlapping cells by the total number of cells in the clusters.^[^ ^72^ ^]^

### Identification of Interregional Clusters

For each cluster, examination of whether any of its cells were located across different brain regions was done. Clusters containing cells that span multiple brain regions were identified as interregional clusters.

### Brain Regions Associated with the Visual System, Somatosensory System, and the Default Mode Network

Based on previous data,^[^ ^46^ ^]^ 7 regions associated with the primate visual system, 22 regions associated with the somatosensory system, and 20 regions associated with the monkey default mode network were collected. Then the correlation between the distribution of neuronal clusters and the characteristics of these different brain regions was analyzed.

### Hierarchical Clustering Analysis of the Cellular Composition Of Excitatory and Inhibitory Neuron Clusters

To explore the characteristics of neuron clusters within cortical layers, a hierarchical clustering analysis was conducted.^[^ ^73^ ^]^ Initially, the proportion of each cell subclass within each cluster was calculated and UMAP dimensionality reduction was utilized to visualize their distribution across layers 1–6. Subsequently, within each layer, hierarchical clustering analysis was performed specifically on excitatory and inhibitory neuron clusters. Binary coding (0 and 1) was used to indicate the absence or presence of specific cell type subclasses in each cluster. To effectively present the results of the hierarchical clustering analysis, the percentage composition of each neuron subclass within each group was displayed.

### Statistical Analysis

Permutation tests were used to evaluate the significance of excitatory and inhibitory neuron clusters, as well as the enrichment of different cell types within each cluster. To compare cluster size and E–I ratios between two groups, the Wilcoxon rank‐sum test was employed. Pearson correlation was utilized to calculate the correlation coefficients. Additionally, chi‐square tests were conducted to determine whether the number of cells in inhibitory neuron clusters was significantly higher than expected. To account for multiple comparisons, p‐values were adjusted using the FDR method.

### Data and Code Availability

All neuronal clusters and their corresponding cell numbers are provided in the supplementary table. All original codes can be made available by the lead contact upon request.

## Conflict of Interest

The authors declare no conflict of interest.

## Author Contributions

G.‐Z.W. conceived and designed the project. R.C. and G.‐Z.W. performed the analysis with assistance from X.N. and L.M. R.C. and G.‐Z.W. wrote the paper with input from all authors.

## Supporting information



Supporting Information

Supplemental Tables 1‐4

## Data Availability

The data that support the findings of this study are available in the supplementary material of this article.
