# Supplementary material for: Organizational Principles of the Primate Cerebral Cortex at the Single‐Cell Level
Source: Adv Sci (Weinh). 2025 Jan 23;12(11):2411041. doi: 10.1002/advs.202411041 (PMC11923899; doi:10.1002/advs.202411041)
Supplement: Supplementary file 1 — Supporting Information [file ADVS-12-2411041-s002.docx]

Supplementary Information for

**Organizational principles of the primate cerebral cortex at the single-cell level**

Renrui Chen, Pengxing Nie, Liangxiao Ma, Guang-Zhong Wang*

**This file includes:**

Figures S1 to S3

Legends for Supplementary Table S1 to S4

**Other Supporting Information for this manuscript includes the following:**

Supplementary Table S1-S4

**Figures S1 to S3**

**
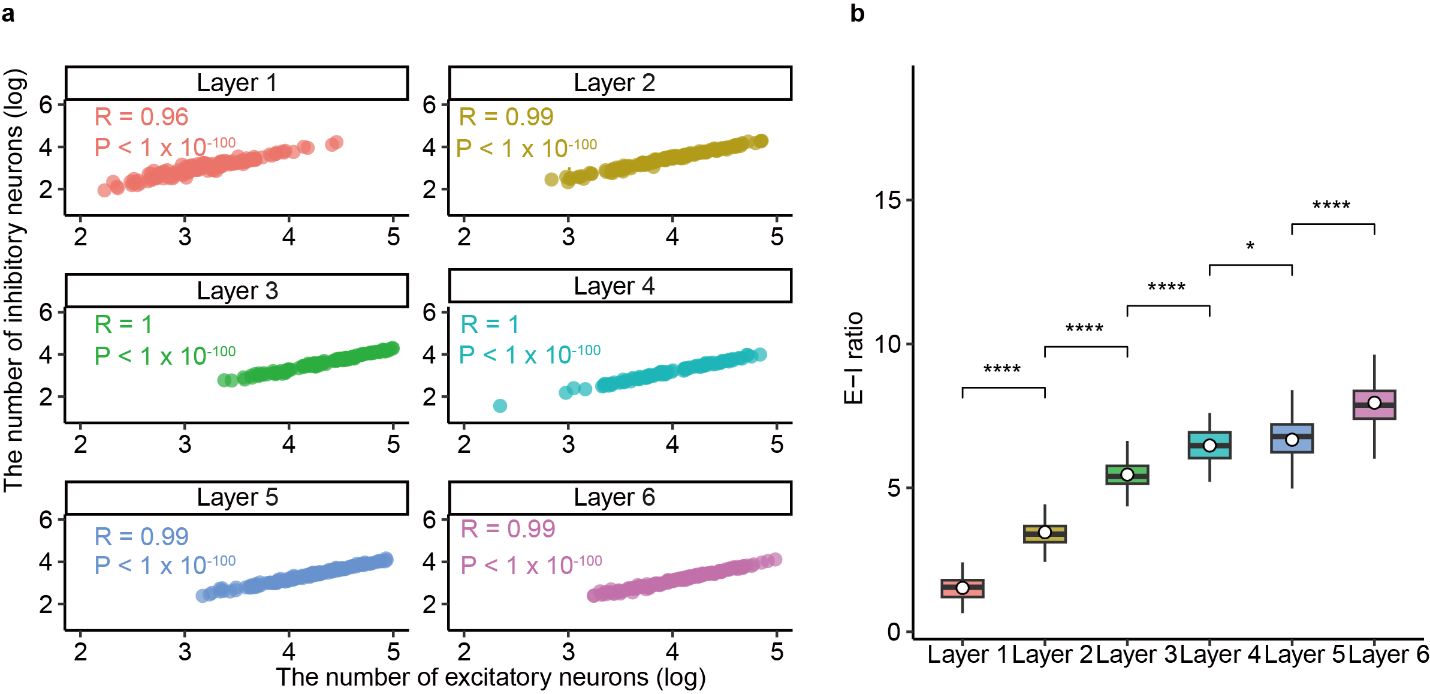
**

**Figure S1. Distribution of excitatory and inhibitory neurons across cortical layers in macaques.** (**a**) Correlation between the number of excitatory and inhibitory neurons across different cortical layers (Layer 1 to 6). Analysis included data from 143 cortical regions of macaque brains. Statistical significance was assessed using Pearson's correlation method. (**b**) Distribution of the excitatory-to-inhibitory (E-I) ratio in each cortical layer, demonstrating a significant increase in the E-I ratio from Layer 1 to 6. Analysis was performed using the Wilcoxon rank-sum test.

**
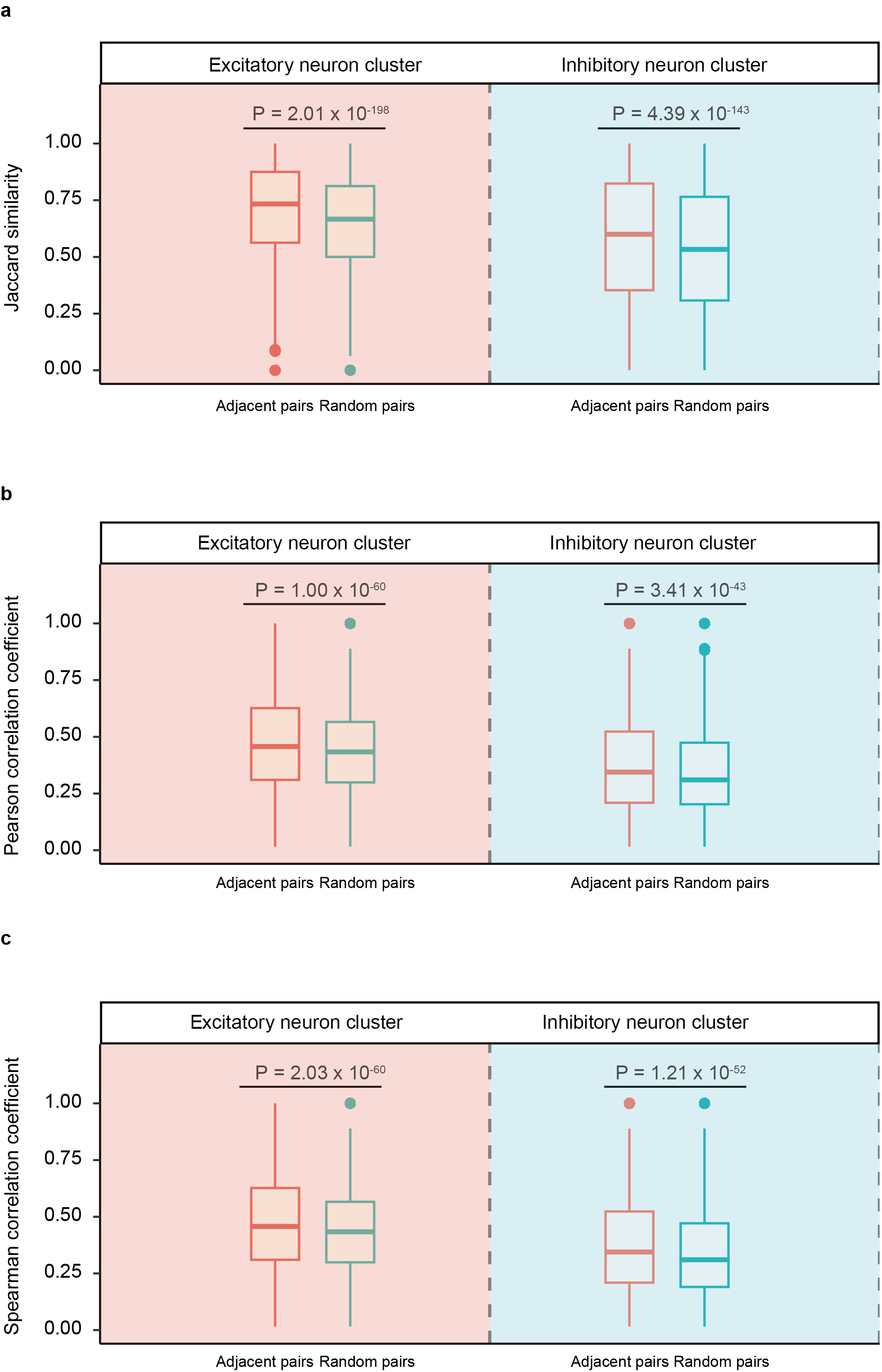
**

**Figure S2. Similarity of cell type usage for adjacent neuron cluster pairs of the same type (either excitatory or inhibitory).** Comparison of the similarity in cell type composition between adjacent neuron clusters (red) and random pairs (blue) using the Jaccard similarity index (**a**), Pearson's correlation coefficient (**b**) and Spearman’s rank correlation coefficient (**c**).

**
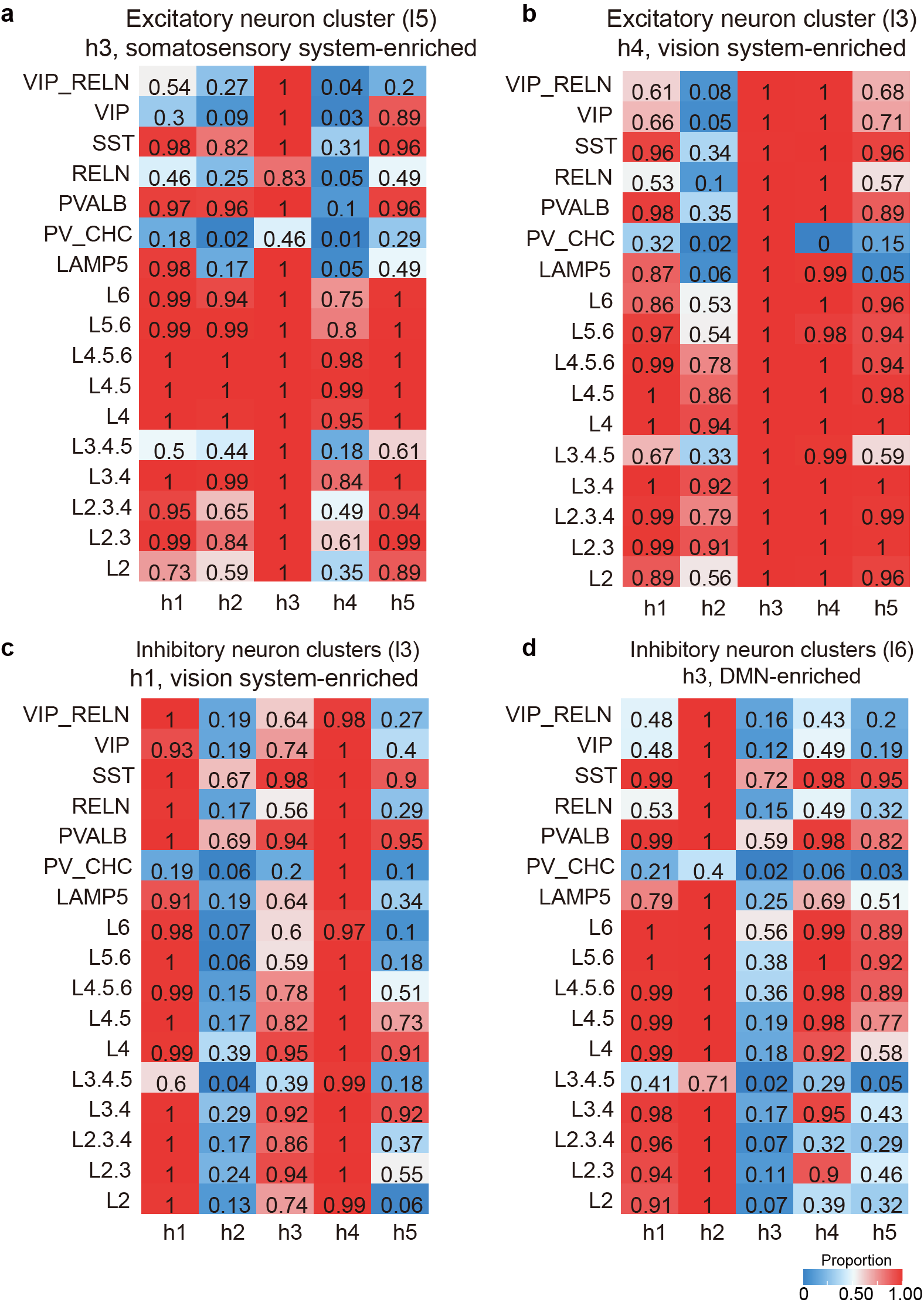
**

**Figure S3. Relationship between neuronal cluster groups and functional characteristics of various brain regions.** This figure illustrates the differences in cell type composition across hierarchical clustering groups. (**a**) Displays excitatory neuron clusters in Layer 5, with group h3 predominantly enriched in the somatosensory systems. (**b**) Shows excitatory neuron clusters in Layer 3, with group h4 primarily enriched in the visual systems. (**c**) The heatmap represents inhibitory neuron clusters in Layer 3, with group h1 significantly enriched in the visual systems. (**d**) The heatmap for inhibitory neuron clusters in Layer 6 identifies group h3 as being enriched in the DMN. In the heatmaps, values closer to 1 indicate a higher prevalence of the cell type within that group.

**Legends for Supplementary Tables S1 to S4**

**Table S1.** Basic information of neuronal clusters.

**Table S2.** Details of cell type usage in neuronal clusters.

**Table S3.** Detailed partnerships between excitatory and inhibitory neuronal clusters.

**Table S4.** Enrichment analysis of hierarchical clustering of neuronal clusters for DMN, visual system, and somatosensory system-related brain regions.
